# Healthcare Utilization of Complex Chronically Ill Children Managed by a Telehealth-Based Team

**DOI:** 10.3389/fped.2021.689572

**Published:** 2021-06-16

**Authors:** Lindsay Braun, Martina Steurer, Duncan Henry

**Affiliations:** ^1^Department of Pediatrics, University of California, San Francisco, San Francisco, CA, United States; ^2^University of California, Benioff Children's Hospital, San Francisco, San Francisco, CA, United States

**Keywords:** complex chronic care, telehealth, technology dependent, care coordination, children with medical complexity

## Abstract

**Objectives:** Medical advances have improved survival of critically ill children, increasing the number that have substantial ongoing care needs. The first aim of this study was to compare healthcare utilization of children with complex chronic conditions across an extensive geographic area managed by a predominantly telehealth-based team (FamiLy InteGrated Healthcare Transitions—FLIGHT) compared to matched historical controls. The second aim was to identify risk factors for healthcare utilization within the FLIGHT population.

**Methods:** We performed a retrospective cohort study of all patients enrolled in the care management team. First, we compared them to age- and technology-based matched historic controls across medical resource-utilization outcomes. Second, we used univariable and multivariable linear regression models to identify risk factors for resource utilization within the FLIGHT population.

**Results:** Sixty-four FLIGHT patients were included, with 34 able to be matched with historic controls. FLIGHT patients had significantly fewer hospital days per year (13.6 vs. 30.3 days, *p* = 0.02) and shorter admissions (6.0 vs. 17.3 days, *p* = 0.02) compared to controls. Within the telehealth managed population, increased number of technologies was associated with more admissions per year (coefficient 0.90, CI 0.05 – 1.75) and hospital days per year (16.83, CI 1.76 – 31.90), although increased number of complex chronic conditions was not associated with an increase in utilization.

**Conclusion:** A telehealth-based care coordination team was able to significantly decrease some metrics of healthcare utilization in a complex pediatric population. Future study is warranted into utilization of telemedicine for care coordination programs caring for children with medical complexity.

## Introduction

Children with medical complexity (CMC) comprise only a small fraction (0.5%) of the pediatric population but have a disproportionate share of healthcare expenditures associated with their care (1). Estimates suggest that CMC account for an increase of 33% of total healthcare expenditures for children in the US as well as 40% of hospital charges (1–3). Costs of care for this populations are in part driven by increasing hospitalization rates (particularly in intensive care units), longer duration of admission, high emergency room utilization, and more frequent readmissions (2–9). These patients often have one or more complex chronic conditions (CCCs), operationally defined by Feudtner as a medical condition that lasts for >12 months and involves several different organ systems or one organ system requiring a high level of specialty care and hospitalization (10). The complexity of their conditions and involvement of multiple teams makes planning their discharge from the hospital and maintaining coordinated care as an outpatient a process that is susceptible to errors and is labor intensive. Outpatient care for this patient population often requires coordination to support new or evolving medical technology, complicated medication regimens, and attending to frequent multi-specialty follow-up.

To address the multitude of issues facing this population, a variety of programs have been developed to address complex care needs (11–18) of these patients. Despite broad variability in their care delivery paradigms, the majority of programs focus on care coordination designed to address not only the medical but also the developmental, emotional, social, and financial needs of children and families (19). A multicenter cohort analysis recently showed a decrease in spending of 4.6% for complex patients enrolled in care management programs, highlighting not only the clinical importance but also the cost effectiveness of such care coordination teams (20). Nonetheless, it is not clear what the optimal design (or designs) for such programs should be, or how best to assess the ability of complex care models to produce meaningful improvements in outcomes (21). In a recent national, multi-stakeholder survey regarding research priorities for CMC, both social determinants of health (including rurality) and clinical model refinement (including telemedicine) achieved highest priority (22).

In 2015, the University of California San Francisco (UCSF) developed a novel care coordination team to serve CMC across the large geographical region of northern California. The FLIGHT (FamiLy InteGrated Healthcare Transitions) team is a multidisciplinary team that provides care coordination and complex care for CMC and their families. FLIGHT is novel in its use of a predominantly telemedicine-mediated format for complex care. To date, very few other programs with this type of structure, serving patients with such high complexity, have been described and there is limited data for patients participating in these programs.

This study aimed to compare FLIGHT participants with matched historical controls with regard to medical resource utilization. Our second aim was to examine risk factors for association with increased medical resource usage within the FLIGHT population.

## Methods

We performed a retrospective chart review study including all 64 patients enrolled in the FLIGHT program at UCSF Benioff Children's Hospital since its start in July 2015.

Inclusion in the program is based on complexity (four or more active subspecialists involved in care) and use of technology at home (examples include non-invasive positive pressure ventilation, tracheostomy, enteral feeding tube), similar to other structured complex care programs (9). Family caregivers, through referrals from inpatient services and outpatient primary care and specialty practices, are approached about the program, provided a description of its services, and afforded an opportunity to accept or decline enrollment. The majority of patients have been referred during or shortly after a neonatal or pediatric intensive care unit admission. FLIGHT is comprised of two part-time physicians—one pediatric intensivist who is the medical director and one volunteer faculty who is board certified in pediatrics and internal medicine. In addition, the FLIGHT team is also comprised of a program administrator responsible for scheduling and triage of calls, one full-time and one part-time case manager, and one part-time social worker. Enrollment is capped at 60 patients given limited resources. Care is provided through an initial in-person consultation as well as quarterly structured telehealth visits *via* a secure, HIPAA-compliant interface (Zoom Video Communications, San Jose, CA, USA). Video visits follow a structured format to accomplish the following: identify and triage medical concerns; review current supplies and needs for durable medical equipment; review, refill, and appropriately route medication requests; identify financial or support needs; ensure enrollment in and/or engage in advocacy for community-based services; and provide family caregiver education regarding technology, logistics, and care coordination. Attempts were made to ameliorate access and connectivity issues to assist the families in accessing telehealth-based services in the following ways: donations of used iPads (Apple, Cupertino, CA, USA) were secured for distribution to families with needs, grant-based assistance to establish a reliable internet connection in the home, and partnership with local home care agencies, public health nurses, and hospice/palliative care services allowing connection through these agencies' devices. Whenever possible, FLIGHT partnered with local home care agencies, public health nurses, and hospice/palliative care services to assist with connection through use of phones/tablets/computers that were available to these agencies. The program administrator assessed connectivity with the family prior to the initial scheduled telehealth appointment.

To address our first aim, we used a case–control design to evaluate the impact of FLIGHT on healthcare utilization compared to historic controls. We used the Virtual PICU Systems (VPS) database (myvps.org) to find historical controls within our institution not enrolled in the FLIGHT program. We extracted all VPS admissions at our institution with one or several of the following technologies present on admission between January 2012 and December 2018: tracheostomy, invasive mechanical ventilation, non-invasive mechanical ventilation, or central line. The technology was defined as present on admission in the VPS database, thus affording us the opportunity to identify technologies that are used at home rather than initiated during the admission. We were unable to use feeding tube as a matched technology as data about this technology is not routinely entered by our institution. As our institution transitioned to electronic medical records in 2011, the year 2012 was selected as the start date to ensure that all patient data was accessible. We matched FLIGHT cases with VPS controls based on age and technology; age was categorized as 0–1 years, 2–4 years, 5–9 years, 10–14 years, and 15 and older. Controls were exactly matched on age category as well as the following technologies: tracheostomy, invasive ventilation, non-invasive positive pressure ventilation, and central line. If FLIGHT patients were selected as a control, only data prior to their enrollment in FLIGHT was collected as control data. Patients who were enrolled in FLIGHT during birth hospitalization were not eligible as controls. Based on these criteria, we were able to match 37 out of 64 of the FLIGHT patients to controls from the VPS database, with the remaining FLIGHT patients unable to find matches in the historical control population.

For the second aim, we performed analyses to explore potential variables associated with high utilization rates within the entire FLIGHT population of patients (*n* = 64). We collected detailed demographic and clinical data from the electronic medical record system. Median income, percent below poverty, and percent with educational attainment high school graduate or above were obtained from American Community Survey 2018 data by linking with the patient's zip code.

As our outcomes, we defined five markers of healthcare utilization: hospital admissions per year, hospital days per year, hospital days per admission, subspecialty appointments per year, and missed appointments per year. These outcomes were compared between the 37 matched FLIGHT patients with the 37 control patients. Dichotomous or categorical variables are presented as n and percentage and compared using chi-square tests. Continuous characteristics are presented as median and interquartile range (IQR) and compared using Wilcoxon rank-sum tests.

We then evaluated all 64 FLIGHT patients for factors associated with increased healthcare utilization, as defined by the outcomes above. Predictors collected from the medical record were age, sex, white vs. non-white race, English as a primary language vs. non-English primary language, public vs. private insurance, number of technologies, and number of CCCs. Predictors collected from ACS 2018 data by linked zip code were median household income, percent education attainment of high school or greater, and percent of individuals below poverty level. Distance from home zip code to hospital zip code was collected from https://www.freemaptools.com/distance-between-usa-zip-codes.htm. We first performed univariable linear regressions for each of our five outcomes. For the multivariable regressions, we controlled for age, number of technologies, and number of CCCs. Results are expressed as coefficients and 95% confidence intervals (CI). A *p*-value < 0.05 was considered statistically significant for all variables. Stata was used for all analyses (StataCorp. 2017. *Stata Statistical Software: Release 15*. StataCorp LLC, College Station, TX, USA). The study was approved by the UCSF institutional reviewing board.

## Results

**Figure 1:**
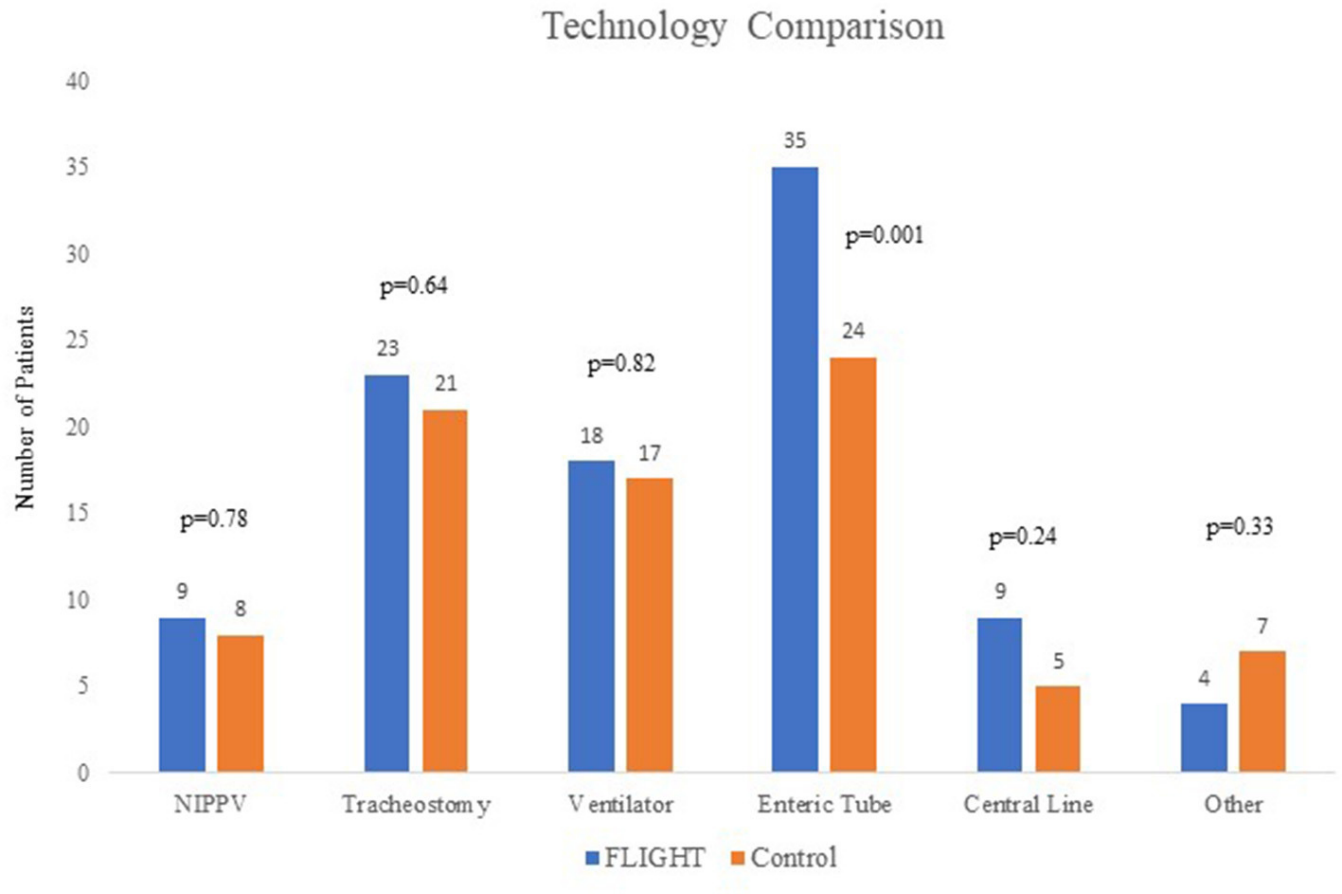
Technology dependence of FLIGHT and control patients.

In total, there have been 64 FLIGHT patients, enrolled between January 2016 and January 2019. Thirty-seven FLIGHT patients were able to be matched to 37 historical controls based on age and technology. As expected, in the matched cohort, there was no difference between (mean) age [5.62 years (SD 7.22) vs. 5.51 years (SD 7.67), *p* = 0.95] or median number of technologies [2 (IQR 2-3) vs. 2 (IQR 1-3), *p* = 0.15] between cases and controls. The FLIGHT group had more CCCs (*p* = 0.04) and a higher percentage of patients on public insurance (89 vs. 70%, *p* = 0.04) than the control group. The number of patients in the FLIGHT group and the controls with each technology (non-invasive positive pressure ventilation, tracheostomy, invasive ventilation, enteric feeding tube, central line, and other) are shown in Figure 1. Enteric feeding tubes were unable to be matched using our VPS data, and there were 35 in the FLIGHT group vs. 24 in the control group, *p* = 0.001. The historical controls had a longer period of data collection (2.95 vs. 0.88 years, *p* ≤ 0.001) than the FLIGHT cases. Age, sex, non-white race, and English as primary language were not different (Table 1). Census data based on the home zip codes of the patients, including median income, percent of population that has completed high school or greater, and percent living below poverty level, were not different, nor was distance to UCSF Benioff Children's Hospital (Table 1).

**Table 1:** Baseline characteristics of FLIGHT and Control patients.

	**FLIGHT (*n* = 37)**	**Controls(*n* = 37)**	***p*-value**
Age in years, mean (SD)	5.6 (7.2)	5.5 (7.7)	0.95
Female sex, *n* (%)	12 (32%)	18 (49%)	0.16
White race, *n* (%)	9 (24%)	13 (35%)	0.31
English speaking, *n* (%)	27 (73%)	31 (84%)	0.26
Public insurance, *n* (%)	33 (89%)	26 (70%)	0.04^*^
Nr. of technologies, median (IQR)	2 (2–3)	2 (1–3)	0.15
Nr. of CCCs, median (IQR)	5 (5–6)	5 (4–6)	0.04^*^
Median household income in zip code in dollars, median (IQR)	63,848 (51,918–84,269)	77,222 (51,918–91,802)	0.30
Percent education attainment high school graduate or higher in zip code, median (IQR)	83.4 (75.7–88.7)	87.1 (78.1–90.2)	0.15
Percent of individuals below poverty level in zip code, median (IQR)	14.9 (8.4–19.7)	10.6 (7.3–18.8)	0.29
Length of data collected in years, median (IQR)	0.9 (0.5–1.4)	3.0 (1.1–4.5)	<0.001^*^
Distance to BCH SF in miles, median (IQR)	67 (21–96)	49 (19–92)	0.47

*CCCs, complex chronic conditions; BCH SF, UCSF Benioff Children's Hospital San Francisco*.

* *p-value < 0.05*.

When comparing the healthcare utilization outcomes between FLIGHT cases and matched controls, we found that FLIGHT patients had significantly fewer hospital days per year [13.60 (IQR 0–52.42) vs. 30.30 (IQR 10.18–142.28), *p* = 0.02] and hospital days per admission compared to controls [6 (IQR 4.10–12.14) vs. 17.33 (IQR 7–28), *p* = 0.02] (Table 2). FLIGHT patients also had significantly more subspecialty appointments per patient year [12.53 (IQR 7.28–20.98) vs. 7.62 (IQR 2.81–10.50), *p* = 0.01] (Table 2). Hospital admission per year and missed outpatient appointments per year did not differ between the two groups.

**Table 2:** Healthcare utilization comparing FLIGHT cases and matched controls.

	**FLIGHT (*n* = 37)**	**Controls(*n* = 37)**	***p*-value**
Admissions per patient per year, median (IQR)	2.0 (0–4.5)	1.9 (0.9–3.9)	0.58
Hospital days per year, median (IQR)	13.6 (0–52.4)	30.3 (10.2–148.3)	0.02^*^
Hospital days per admission, median (IQR)^a^	6 (4.1–12.1)	17.3 (7–28)	0.02^*^
Number of subspecialty appointments per year, median (IQR)	12.5 (7.3–21.0)	7.6 (2.8–10.5)	0.01^*^
Missed appointments per year, median (IQR)	0.4 (0–2.6)	1.0 (0–1.4)	0.45

a *Excluded FLIGHT patients with no hospital admissions during data collection*.

* *p-value < 0.05*.

We examined predictors for increased healthcare utilization in the entire FLIGHT population (*n* = 64). Table 3 shows the results of the univariable linear regression analyses evaluating associations of predictors selected *a priori* with increased medical resource utilization. Number of technologies was associated with more admissions per year (*p* = 0.04) and hospital days per year (*p* = 0.03), but number of CCCs was not associated with increased medical resource utilization. Table 4 shows the results of the multivariable model. Each model was controlled for age, number of technologies, and number of CCCs. Each one-unit increase in number of technologies was associated with more admissions per year (coefficient 0.90 admissions, CI 0.05–1.75) and hospital days per year (coefficient 16.83, CI 1.76–31.90).

**Table 3:** Univariable models for different risk factors for increased healthcare utilization in the FLIGHT patient group (*n* = 64).

**Predictor**	**Admissions per year**	**Hospital days per year**	**Hospital days per admission**	**Subspecialty appointments per year**	**Missed subspecialty appointments per year**
	**Coefficient (*p*-value)**	**Coefficient(*p*-value)**	**Coefficient (*p*-value)**	**Coefficient(*p*-value)**	**Coefficient (*p*-value)**
Age (yrs)	−0.03!!break (0.67)	0.11(0.92)	0.35!!break (0.34)	−0.22(0.24)	0.03!!break (0.70)
Sex (female vs. male)	−1.77!!break (0.03)^*^	−26.99(0.06)	−4.78!!break (0.30)	−1.12(0.65)	−1.04!!break (0.23)
Race (white vs. non-white)	0.81!!break (0.36)	2.55(0.87)	−3.62!!break (0.43)	1.70(0.52)	0.23!!break (0.81)
English speaking (yes vs. no)	−0.34!!break (0.97)	11.83(0.49)	3.35!!break (0.53)	1.36(0.64)	0.17!!break (0.87)
Nr. of technologies	0.93!!break (0.02)^*^	15.26(0.03)^*^	1.34!!break (0.58)	0.11(0.93)	−0.10!!break (0.82)
Nr. of CCCs	0.42!!break (0.25)	2.77(0.67)	0.05!!break (0.98)	0.49(0.66)	−0.23!!break (0.56)
Public insurance (yes vs. no)	0.55!!break (0.59)	−13.78(0.45)	−8.04!!break (0.15)	3.15(0.31)	−1.14!!break (0.29)
Presence of home nursing (yes vs. no)	1.68!!break (0.06)	14.39(0.36)	−0.18!!break (0.97)	0.06(0.98)	−0.85!!break (0.36)
Median household income of zip code^a^	0.008!!break (0.55)	0.23(0.36)	0.06!!break (0.53)	0.01(0.75)	−0.01!!break (0.37)
Percent of zip code with education high school or above	0.07!!break (0.04)^*^	0.87(0.14)	0.07!!break (0.73)	0.11(0.26)	−0.01!!break (0.69)
Percent of zip code below poverty level	−0.02!!break (0.64)	−0.79(0.36)	−0.42!!break (0.26)	−0.05(0.73)	0.03!!break (0.62)
Distance to BCH SF	−0.001!!break (0.82)	−0.01(0.94)	0.001!!break (0.97)	−0.01(0.33)	0.004!!break (0.44)

*CCCs, complex chronic conditions; BCH SF, UCSF Benioff Children's Hospital San Francisco*.

* *Values are significant with a p-value < 0.05*.

a *Results are presented by increase of $1,000 of the median household income of the zip code, i.e., for each increase in $1,000 household income, there are 0.008 less admissions per year*.

**Table 4:** Associations between five measures of healthcare utilization in the FLIGHT patient group (*n* = 64) and patient characteristics controlling for age, number of technologies, and number of CCCs.

**Predictor**	**Admissions per year**	**Hospital days per year**	**Hospital days per admission**	**Subspecialty appointments per year**	**Missed subspecialty appointments per year**
	**Coefficient (*p*-value)**	**Coefficient(*p*-value)**	**Coefficient (*p*-value)**	**Coefficient(*p*-value)**	**Coefficient (*p*-value)**
Age (yrs)	−0.02 (0.81)	0.16(0.88)	0.34 (0.38)	−0.21(0.28)	0.02 (0.80)
Sex (female vs. male)	−1.53 (0.07)	−21.79(0.14)	−5.44 (0.26)	−0.99(0.71)	−1.12 (0.23)
Race (white vs. nonwhite)	1.13 (0.20)	4.5(0.78)	−4.47 (0.36)	2.48(0.36)	0.10 (0.92)
English speaking (yes vs. no)	−0.18 (0.86)	10.47(0.55)	2.83 (0.61)	1.97(0.52)	0.22 (0.84)
Nr. of technologies	0.90 (0.04)^*^	16.83(0.03)^*^	1.36 (0.62)	−0.09(0.95)	0.002 (0.99)
Nr. of CCCs	0.06 (0.88)	−3.39(0.64)	−0.22 (0.93)	0.25(0.84)	−0.21 (0.64)
Public insurance (yes vs. no)	0.90 (0.38)	−8.72(0.63)	−6.81 (0.26)	3.04(0.34)	−1.21 (0.28)
Presence of home nursing (yes vs. no)	1.39 (0.15)	5.73(0.74)	−2.27 (0.67)	0.98(0.75)	−1.01 (0.34)
Median household income by zip code^a^	0.02 (0.27)	0.33(0.20)	0.06 (0.49)	0.03(0.53)	−0.02 (0.30)
Percent of zip code with education high school or above	0.07 (0.02)^*^	0.95(0.10)	0.09 (0.67)	0.12(0.23)	−0.02 (0.66)
Percent of zip code below poverty level	−0.04 (0.38)	−1.08(0.21)	−0.46 (0.23)	−0.08(0.58)	0.03 (0.57)
Distance to BCH SF	−0.001 (0.81)	−0.003(0.97)	0.00003 (0.99)	−0.01(0.30)	0.004 (0.43)

*CCCs, complex chronic conditions; BCH SF, UCSF Benioff Children's Hospital San Francisco*.

* *Values are significant with a p-value < 0.05*.

a *Results are presented by increase of $1,000 of the median household income of the zip code, i.e., for each increase in $1,000 household income, there are 0.008 less admissions per year*.

## Discussion

This study provides a description of patients enrolled in a complex care telehealth program and their healthcare utilization patterns. More importantly, this study shows that enrollment in this program was associated with decreased healthcare utilization. Interestingly this association persisted despite the fact that known risk factors for healthcare utilization (e.g., number of CCCs) were significantly higher in the FLIGHT population as compared to controls.

We hypothesize that this may be due to several factors. First, the presence of an outpatient complex care team which actively engaged with inpatient discharging teams may provide opportunities to identify and address barriers prior to discharge resulting in reduced hospital days. Programs focusing on discharging ventilator-dependent children (a large proportion of the FLIGHT population) have shown similar improvement on these metrics (23) as has a recent randomized control trial looking at inpatient consultation by outpatient complex care physicians (24). Furthermore, availability of a complex care team to continue to address elements of care initiated in the inpatient environment (e.g., referral to home nursing agencies) could have enhanced the discharging team and family's comfort in ensuring follow-up and attention to complex outpatient needs. Another possibility is that the availability of a complex care team with structured follow-up increased parental comfort and confidence in managing their child's condition at home. Families, particularly in the index discharge home with a medically complex child, have the potential to be overwhelmed by the number of resources and services that they need to coordinate upon return home (25). Simplifying access to specialty care, problem-solving durable medical equipment issues, and having one point of contact may have provided families with a simplified resource to help in the peri-discharge period that allowed more efficient and effective contact with their specialty providers and community supports. Furthermore, a qualitative study of caregivers' perceptions regarding hospital to home discharges for medically complex patients highlighted three domains: caregiver self-efficacy, adequacy of support and resources, and comprehensive knowledge of the care plan (26). These are explicit elements of the care coordination that are provided by the FLIGHT program that may have enhanced families' perceptions of discharge readiness. Finally, local knowledge of community-based resources as well as relationship building with local supports may have also contributed to reduced hospital stays. Through its partnership with families, FLIGHT has built substantive knowledge of and advocacy around enrollment in alternative care programs such as special needs daycare programs, counseling of families regarding options for discharge to long-term care facilities, and continued advocacy for financial and physical supports (e.g., home nursing agencies, public health nurses, county-based developmental supports). Attention to engaging these resources after discharge may have increased a family's capacity to remain at home and feel supported in doing so.

This study builds on evidence from other studies that complex care coordination teams can decrease healthcare utilization (15, 20, 24). In contrast to other programs, our model emphasizes a consultative/co-management strategy and in particular, given our large geographic catchment area, utilizes a telehealth care delivery system almost exclusively for its outpatient follow-up. Although over half of visits were *via* telehealth prior to the presence of COVID-19, since the start of the pandemic >95% of outpatient visits are conducted *via* telehealth (internal data). While the use of telehealth has become more ubiquitous, its use for complex care management particularly with highly complex children has not been thoroughly explored (19). Previous studies evaluating the impact of telehealth in CMC have shown a decrease in unplanned visits (27) and lower hospitalization rates (28), and we hypothesize that the use of regularly scheduled visits allows preventative attention to lapses in care that might exacerbate unplanned admissions and discharge delays. It is also possible that FLIGHT identifies issues earlier, leading to shorter admissions to remedy clinical problems and explaining why a decrease in number of admissions was not seen. Further evaluation of the program will analyze 30-day readmission rates and readmissions after a new technology was added to the patient's home care. Given the geographic area that is served by the FLIGHT team, telehealth visits *via* Zoom® are likely more feasible for families than navigating the logistics of transporting a child and their medical equipment, with adequate supervision for necessary medical procedures, up to 7 h for outpatient appointments. It is interesting to note that given the ongoing COVID-19 pandemic, telehealth-mediated healthcare has been increasingly utilized to deliver care in the home environment. Our study demonstrates, with data prior to the COVID pandemic, at least one aspect of healthcare that can presumably be positively impacted through telehealth-based models of care coordination.

When examining the FLIGHT patients for risk factors for increased medical resource utilization, this study is similar to others in demonstrating the association between increasing number of technologies with higher rates of admission and increased total hospital days (6). In contrast, increasing number of CCCs, within FLIGHT patients, was not associated with increased admissions or more hospital days. This suggests that, within our population, technology was the primary driver for healthcare utilization as opposed to increasing number of complex conditions. One potential outcome from this finding is that programs with limited resources and enrollment capacity may choose to focus on triaging enrollment based on technological usage as opposed to other markers of medical complexity. Moreover, while not powered to look explicitly at these metrics, there were no differences in our measured healthcare utilization based on income, gender, race (although admittedly used in a binary fashion), distance from tertiary care center, or the presence/absence of home nursing. This raises the possibility that care coordination, specifically when we can ensure adequate access to telehealth services including connectivity, may be able to address or ameliorate aspects of healthcare inequity. Having familiarity with many of the technologies used by these children and families, the comfort with utilizing telehealth for assessment, and the ability to function in a consultative role also suggest novel avenues for expansion of pediatric intensivists scope of practice beyond the confinement of the intensive care unit (ICU). Previous studies have shown an association between socioeconomic status and inpatient and ICU days, imaging, and interventions at end of life care in CMC (29, 30). We also know that inequities in healthcare access are prevalent among children (31) and may be exacerbated in children with CCCs. This may be further compounded when such children live significant distances from specialty care services. As such, care delivery models that attend to these barriers deserve further investigation.

This study has a few limitations. The study was conducted retrospectively and has a small cohort size. As FLIGHT is a relatively new program, the data collection period is shorter for the cases than the controls. We addressed this by defining outcomes as per patient year to compare to our controls. Within the VPS database, we were only able to match 37 of the total 64 FLIGHT patients and were unable to match enteric feedings tubes, with 35 FLIGHT patients using feeding tubes compared to only 24 in the control cohort. In addition, within our data set we did not show increased healthcare utilization related to previously recognized risk factors (i.e., number of CCCs), apart from number of technologies. This potentially could be due to the small cohort size and its limited data, although it may also reflect that FLIGHT, in its novel implementation and structure, was successful in addressing previously known risk factors for higher utilization among this population. There may be selection bias in the outcomes of patients who accepted FLIGHT compared to those who did not. However, as only three patients declined participation, we were not able to determine if there were significant differences. We were also unable to examine care received outside of our institution, although given these patients' complexity, it is our experience that they are routinely referred and transferred to their home institution. As we did not have individual socioeconomic data, we linked patients with their zip codes, although acknowledge that this may be associated with inaccuracies as that data does not necessarily reflect the individual patient. With the renewed focus on diversity and equity, we acknowledge that the ability to further characterize results by National Institutes of Health racial categories would be helpful to inform discussion about healthcare equity, but were unable to do so due to the limited size of our data set.

Future study is warranted to investigate the cost of these patients' care, particularly with the utilization of telemedicine for care coordination. Additional granular data to assess for differences in clinical trajectories after admission, including illness severity, ICU length of stay, mechanical ventilation, bedside procedures, and recent addition of home technology such as *de novo* tracheostomies will be analyzed to further assess these complex patients' care and help determine if this type of program can detect clinical deterioration earlier. Qualitative evaluation of caregivers' perspective of the telehealth-based care received although the FLIGHT program is also planned, including lost work days, lost school days, and lost wages. Telehealth-based care coordination programs have the potential to be successful in reducing healthcare utilization (and their associated costs) for the most complex of pediatric patients, and to begin to address and ameliorate previously associated variables that may contribute to healthcare inequities.

## Data Availability Statement

The raw data supporting the conclusions of this article will be made available by the authors, without undue reservation.

## Ethics Statement

Ethical review and approval was not required for the study on human participants in accordance with the local legislation and institutional requirements. Written informed consent from the participants' legal guardian/next of kin was not required to participate in this study in accordance with the national legislation and the institutional requirements.

## Author Contributions

LB conceptualized and designed the project, designed the data collection instruments, collected the data, drafted the initial manuscript, and reviewed and revised the manuscript. MS conceptualized and designed the project, supervised the data analysis, and reviewed and revised the manuscript. DH conceptualized and designed the project and reviewed and revised the manuscript. All the authors approved the final manuscript as submitted and agreed to be accountable for all aspects of the work.

## Conflict of Interest

The authors declare that the research was conducted in the absence of any commercial or financial relationships that could be construed as a potential conflict of interest.
